# Anthropogenic and climatic factors interact to influence reproductive timing and effort

**DOI:** 10.1002/ece3.11306

**Published:** 2024-05-10

**Authors:** Geoffrey D. Smith, Travis E. Wilcoxen, Spencer B. Hudson, Emily E. Virgin, Andrew M. Durso, Marilize Van der Walt, Austin R. Spence, Lorin A. Neuman‐Lee, Alison C. Webb, Patricia A. Terletzky, Susannah S. French

**Affiliations:** ^1^ Department of Biological Sciences Utah Tech University St. George Utah USA; ^2^ Department of Biology Millikin University Decatur Illinois USA; ^3^ Department of Biology Utah State University Logan Utah USA; ^4^ Ecology Center Utah State University Logan Utah USA; ^5^ Department of Biological Sciences Florida Gulf Coast University Ft. Myers Florida USA; ^6^ Department of Wildlife, Fish, and Conservation Biology University of California ‐ Davis Davis California USA; ^7^ Department of Biology Arkansas State University Jonesboro Arkansas USA; ^8^ Department of Wildland Resources Utah State University Logan Utah USA

**Keywords:** fitness, precipitation, reproductive stage, stress, urbanization

## Abstract

Reproduction, although absolutely essential to a species’ persistence, is in itself challenging. As anthropogenic change increasingly affects every landscape on Earth, it is critical to understand how specific pressures impact the reproductive efforts of individuals, which directly contribute to the success or failure of populations. However, organisms rarely encounter a single burden at a time, and the interactions of environmental challenges can have compounding effects. Understanding environmental and physiological pressures is difficult because they are often context‐dependent and not generalizable, but long‐term monitoring across variable landscapes and weather patterns can improve our understanding of these complex interactions. We tested the effects of urbanization, climate, and individual condition on the reproductive investment of wild side‐blotched lizards (*Uta stansburiana*) by measuring physiological/reproductive metrics from six populations in urban and rural areas over six consecutive years of variable precipitation. We observed that reproductive stage affected body condition, corticosterone concentration, and oxidative stress. We also observed that reproductive patterns differed between urban and rural populations depending on rainfall, with rural animals increasing reproductive investment during rainier years compared to urban conspecifics, and that reproductive decisions appeared to occur early in the reproductive process. These results demonstrate the plastic nature of a generalist species optimizing lifetime fitness under varying conditions.

## Graphical abstract

Reproduction changes with precipitation in side‐blotched lizards. However, urban and rural populations are affected differently by precipitation changes. Here we attempt to determine the internal cues that control those changes.

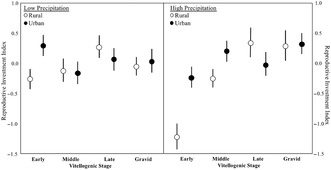

## 1 INTRODUCTION

The great diversity of reproductive strategies that allow organisms to propagate despite environmental challenges demonstrates the selective pressure on this evolutionary imperative. Furthermore, this diversity of reproductive approaches indicates that not all are optimal in any given situation (Ricklefs & Wikelski, 2002), and reproductive options must be selected to enhance reproductive success in response to changing environments. Extrinsic factors such as weather events, fluctuating resources, and anthropogenic changes can affect reproductive timing and viability, and different species are known to utilize different strategies in response (James & Shine, 1985). Similarly, intrinsic factors such as functional constraints that arise from limited resources (e.g., energy, nutrients, time) allocated among competing, internal demands (Gadgil & Bossert, 1970) affect reproductive success. Immediate reproductive investment is therefore determined by the costs that can be afforded with respect to other key fitness traits (Roff et al., 2006). Because selection operates on relative fitness during specific ecological conditions (Ricklefs, 2000), reproductive decisions (i.e., how and when to reproduce) must adjust in variable environments to optimize lifetime fitness (Williams, 1966). Further complicating these patterns is the current pace of human development across the planet, which is impacting the landscapes in which animals reproduce at a scale and rate that many species will not survive (Ceballos et al., 2017; Otto, 2018). It is therefore more critical than ever to understand how different organisms respond to and reproduce in changing environments, what characteristics allow them to be successful, and how reproductive strategies change with changing pressures. These qualities and relationships are inherently multifaceted, phylogenetically constrained, and context‐dependent (thus changing over time), which often makes reproductive patterns difficult to discern and harder to predict. However, the value of better understanding the nuances of individual systems has never been more evident given the rate of global change.

Abiotically, water availability can be a powerful limiting factor for wild animals (Zani & Stein, 2018), but some species have demonstrated physiological trade‐offs that can mitigate or even enhance reproductive success during drought conditions, at least in the short‐term (Dupoué et al., 2018). Furthermore, these environmental characteristics have been shown to interact and drive life histories (Wang et al., 2016). Abiotic conditions also drive biotic pressures. Pettigrew and Bull (2012) demonstrated that drought conditions (e.g., less vegetative cover) resulted in pygmy bluetongue lizards (*Tiliqua adelaidensis*) exhibiting more frequent bold predation attempts, a behavior that could increase the likelihood of their own predation. Conversely, reduced activity and subsequent predation was observed in side‐blotched lizards (*Uta stansburiana*) during droughts (Wilson, 1991). Aside from predation, interspecific competition can increase in response to water limitation (Dunham, 1980), but intraspecific competition for territory can be more complex (Stamps, 1987). The great diversity of physiological and behavioral traits that help ensure reproductive success, even in the face of suboptimal environmental conditions, indicates they have evolved in many specific organisms in many specific habitats, but those habitats are changing quickly. The rapid anthropogenic changes impacting the world and interacting with the aforementioned pressures serve as relatively new challenges to organisms and their reproductive success and should be considered.

Human activities have affected every aspect of ecology on earth (Crutzen, 2006; Steffen et al., 2007). Although overexploitation and poaching certainly contribute (Branch et al., 2013; Rosser & Mainka, 2002), as does global climate change (Cahill et al., 2013; Thomas et al., 2004), the largest driver of human‐caused extinctions worldwide is habitat destruction and degradation (Pimm & Raven, 2000). Urbanization is a powerful driver of habitat change (McKinney, 2006), and has been estimated to be the largest threat to biodiversity in the United States (Czech et al., 2000). Until recently, there was a paucity of empirical evidence linking anthropogenic disturbance, including urbanization, with physiological parameters, survival, and, ultimately, fitness (Wikelski & Cooke, 2006). However, evidence regarding the direct impacts of urbanization on the physiology, reproduction and survival in wildlife is amassing (French et al., 2018). Changes in temperature, light, noise, water, and introduction of non‐native species associated with urban environments are emerging as significant challenges to wildlife (Davies et al., 2017; Gaertner et al., 2017; Lertzman‐Lepofsky et al., 2020; Malisch et al., 2020; Ouyang et al., 2018). For example, artificial light accelerated reproduction in European blackbirds (*Turdus merula*) (Dominoni et al., 2013), and altered timing and peaks in reproductive hormones in tree sparrows (*Passer montanus*) (Zhang et al., 2014). Anthropogenic disturbance and temperature interact to influence breeding phenology in the common lizard (*Zootoca vivipara*) (Rutschmann et al., 2016), and urban heat island temperatures affect development rate of embryos in Puerto Rican crested anole lizards (*Anolis cristatellus*) (Hall & Warner, 2018). It soon becomes evident that multiple factors within urban settings can interact to influence wildlife physiology, and, most significantly, reproduction. However, not all animals are negatively impacted by urban environments. In fact some species, termed urban exploiters, thrive under anthropogenically altered conditions (Blair, 1996). Although urban exploiters (and to a lesser degree, urban adapters) have been found to share some characteristics that allow them to persist and take advantage of novel environments, they do not necessarily have a single trait in common, but rather a suite of behavioral and physiological traits, including diet, nesting preference, sociality, and more, which allow them to flourish (Kark et al., 2007). Understanding how these individual characteristics interact is an important and oft‐missing key to understanding why some populations persist and others fail as environmental conditions change, be it naturally, anthropogenically, or more likely, combinations of the two.

Finally, the degree to which external perturbations impact reproduction may depend on their point in the reproductive cycle. Physiological attributes can vary widely with reproductive stage, and energetic demands from reproduction are different between early and late stages. In painted dragon lizards (*Ctenophorus pictus*), yolk precursors were more highly allocated at the onset of vitellogenesis compared to later stages (Lindsay et al., 2020), and circulating plasma metabolites were highest during early‐vitellogenesis in Colorado checkered whiptails (*Aspidoscelis neotesselatus*) (Hudson et al., 2020), suggesting temporal differences in investment or available energy. Similarly, female great tit (*Parus major*) resting metabolic rates were higher during egg‐formation than other reproductive stages such as nest building and chick feeding (Nilsson & Råberg, 2001). If available energy and needs differ across reproductive stages, then the response to internal and external stressors must differ as well. For instance, female side‐blotched lizards altered immune investment in response to a wound depending on what stage of vitellogenesis they were in (Pettit et al., 2019), and pregnant pygmy rattlesnakes (*Sistrurus miliarius*) exhibited an altered response to a lipopolysaccharide immune challenge relative to non‐reproductive and male rattlesnakes (Lind et al., 2020).

Given the dramatic changes across an animal's entire physiology during reproduction, the impact of stressors could also depend on reproductive investment, and some responses to challenges could be beneficial for earlier reproductive stages, but not later ones. These responses are mediated by internal signals (e.g., hormones and cytokines) which can help us better understand the internal decision‐making process and the physiological costs incurred by those reproductive choices. For instance, glucocorticoids are released in response to external stressors (Sapolsky et al., 2000; Wingfield et al., 1998) and are thought to mediate physiological tradeoffs via their ability to divert resources towards different physiological functions, including reproduction (French, Johnston, & Moore, 2007; French, McLemore, et al., 2007; Romero, 2002; Wilson & Wingfield, 1992). Glucocorticoids are expected to be highest during energetically demanding life history stages, such as breeding (Romero, 2002) and lactation (Zhang et al., 2020). In addition, current work shows dramatic changes in markers of oxidative stress, which is defined as the imbalance of reactive oxygen species (ROS) and antioxidants (Sies & Jones, 2020). Both reproductive effort (Webb et al., 2019) and glucocorticoid levels (Costantini et al., 2011; Stier et al., 2009; Zafir & Banu, 2009) have been linked to oxidative stress. It is possible that the interplay among these traits is part of the suite of characteristics that determines an organism's response, and perhaps success, in shifting environments.

The present study assessed six consecutive years of individual health metrics, reproductive investment, and stress measures across six different populations of side‐blotched lizards spanning an urban–rural landscape matrix in or near Saint George, Utah, USA, one of the fastest‐growing urban areas of the United States. Side‐blotched lizards (*U. stansburiana*) represent an ideal system to address these key gaps in understanding the reproductive process in free‐living animals. They are small, short‐lived (<4 years old for the current study), abundant, and found across a variable urban–rural landscape, and reproductive investment and stage can be readily assessed via palpation or ultrasonography (Gilman & Wolf, 2007). To accurately assess baseline glucocorticoid levels, which is important in environmental endocrinology (Bonier et al., 2009), blood samples must be collected rapidly (Lucas & French, 2012; Moore et al., 1991; Romero, 2002), which can be challenging in many species. Side‐blotched lizards are an attractive model due to the speed and ease of handling, and the rapidity with which physiological samples can be obtained in a minimally invasive manner. Specifically, we measured clutch size and follicular length in females using a high‐resolution ultrasound, body condition, reactive oxygen species (d‐ROMs) as an indicator of oxidative stress, baseline corticosterone levels, and bacterial killing ability to assess immunity. To investigate environmental influence, we quantified anthropogenic disturbance using spatial analysis of study sites and incorporated annual precipitation. We integrated these measures into a model selection framework using mixed models to address the following questions: (1) Within individuals, how do condition and physiological factors interact with the environment to affect reproductive investment across reproductive stage? (2) How does annual variation in precipitation affect reproductive investment at urban and rural sites?

Although ecological systems are inherently complex within a single year, and multiple years with varying abiotic conditions are even more complicated, we had several assumptions that led to predictions. First, males and females will respond differently, and given the physiologically demanding pressures of vitellogenesis, the factors contributing to female reproductive investment will be more complex, varying with vitellogenic stage. Spermatogenesis is less energetically expensive than vitellogenesis, and males do not pass through vitellogenic stages. We predict not only that males and females will respond differently, but that the explanatory models will be more complicated for the females.

Second, we predict some factors will affect both males and females, specifically precipitation and site type (urban or rural). Precipitation will affect reproductive outcomes, likely leading to greater reproductive investment in wetter years (Padilla & Angilletta, 2022), and site type (urban or rural) will also affect reproductive outcomes in both sexes. Further, we expect the effect of precipitation to be different at different site types. While the two‐way interaction of precipitation and site type might have enough explanatory power for males, the more complicated reproductive effort in females will likely require more variables and potential interactions.

Finally, in females, we predict individual physiological factors (oxidative stress, immunocompetence, and/or hormone levels) will also affect reproductive investment, e.g., oxidative stress and reproductive investment in females may correlate negatively (Agarwal et al., 2012). Further, precipitation and site type may interact with a physiological factor, with potential three‐way interactions involving physiological attributes such as oxidative stress, immunocompetence, or corticosterone levels. For instance, a drought year might affect urban populations less severely than rural populations due to supplemental watering for landscape, which could keep the invertebrate community healthy enough to buffer the drought (two‐way interaction). However, female lizards in a specific vitellogenic stage or other physiological condition might not be able to take full advantage of available resources due to internal constraints, such as high oxidative stress which could lead to anorectic behavior even if resources are abundant enough (three‐way interaction).

## 2 MATERIALS AND METHODS

### 2.1 Study sites

We sampled sexually mature male (*n* = 472) and female (*n* = 445) side‐blotched lizards from six locations within and around Saint George, Utah, USA, in the last week of April or the first 2 weeks of May from 2012 to 2017 (Table 1). For the past 5 years, the U.S. Census Bureau has recognized the Saint George metropolitan area as among the fastest growing in the United States, and the town is currently ranked 6th with a human population growth rate of 3.1%. The established urban center, along with the quickly expanding sprawl, presents researchers with a prime model for studying urbanization. Three of the sites were patch habitats located within Saint George. They were exposed daily to human impact in the form of recreational activities (such as jogging and trail running), as well as construction and maintenance activities. The three rural sites were located in the Dixie National Forest, and were not exposed to such human pressures. The time of year was chosen in attempt to assess animals yolking their first clutch of the year, and to avoid any potential issues with subsequent clutches having different reproductive effort.

**TABLE 1:** Sample size of male and female lizards captured from 2012 to 2017 at rural and urban study sites.

Year and sex	Rural	Urban
2012 Females	22	22
2012 Males	32	28
2013 Females	30	31
2013 Males	27	30
2014 Females	41	31
2014 Males	30	35
2015 Females	56	52
2015 Males	59	48
2016 Females	42	31
2016 Males	59	33
2017 Females	49	38
2017 Males	53	38

### 2.2 Precipitation

We quantified annual precipitation across the study period using data generated by the Saint George Airport weather station (KGSU) and obtained from Weather Underground (http://www.wunderground.com). Due to variation in exact precipitation values across sites and a single regional weather station not directly connected with one of the study sites, we calculated the average precipitation across the 6 years of the study and consolidated annual precipitation into wet (17.56 ± 1.69 cm) and dry (10.89 ± 0.58 cm) years based on whether they were above or below the mean. Average temperature for the month of May varied from 19.87°C to 24.32°C, but the temperature data from the nearest weather station were incomplete and further analysis of maximum and minimum temperatures was not possible.

### 2.3 Spatial analysis of human disturbance

No consistent measures of human disturbance were available across the study areas over time, so we developed a spatially quantified measure of human impacts at each study site. Study sites were represented as areas sampled, including a surrounding buffer of 250 m. The boundaries of each study site, urban structures, trails, roads, and water sources were digitized from 2013, 2015, and 2017 Google Earth imagery (Source: Google Earth accessed October 7, 2017). Man‐made urban structures were identified by the specific function when possible (e.g., roads, houses, parking lots were labeled as such). Identification of trails and roads was based on a combination of the first author's field experience and visible paths in the remotely sensed imagery. All digitized features were projected to Universal Transverse Mercator (UTM), WGS84 datum, and zone 12 N to facilitate valid measures of areas and distances. All imagery processing and feature manipulations were conducted in ArcMap 10.5 (ESRI, Redlands, CA, USA).

We quantified the number and size of urban features and the number and distance of trails and roads that intersect each study site. Distances were measured between the study site and the nearest urban feature. Distances between study sites and polygonized features (e.g., houses, roads) were measured between the two closest edges of each polygon. Distances of zero indicated the feature was within the site area. We quantified the area of urban features present within each study site with a Wilcoxon signed‐rank test to ensure the urban sites were truly different than the rural sites (*p* = .049).

### 2.4 Field capture, measurements, and samples

We captured all individuals with a snare pole between 08:00 and 13:00 with the consideration that lizard activity and corticosterone levels exhibit daily fluctuations (Choudhury et al., 1982). Within 3 min of each capture, we collected blood samples from the retro‐orbital sinus using a heparinized capillary tube. By collecting blood samples within this timeframe, we were able to determine baseline corticosterone concentrations, which is related to the animal's current energetic and stress level (Lucas & French, 2012). We stored blood samples on ice until further processing could take place upon conclusion of daily collection, at which time we separated the plasma from cells via centrifugation and stored the samples at −20°C.

Upon capture, we toe‐clipped and marked individuals with a unique 4‐digit code and marked their dorsal surfaces with non‐toxic paint pen for visual identification. We determined sex, mass (in g using a digital balance or handheld Pesola scale), and snout‐vent length (SVL, in mm). By regressing SVL on body mass and calculating the residuals, we provided a body condition score for each individual. For females, we additionally scored vitellogenic stage based on follicular size and firmness via manual palpation following French, Johnston, and Moore (2007), counted the number of follicles in their current clutch, and measured total follicular length (summed across follicles) via ultrasonography (MicroMaxx, SonoSite, Bothell, WA, USA). Upon completion of all sampling and data collection, we returned individuals to their point of capture. All animals were collected following guidelines provided by the Utah Division of Wildlife Resources (COR 1COLL8382) and all experimental procedures were approved by Utah State University IACUC (protocol 2068). Our data come from individual observations, i.e., these observations do not come from individuals repeatedly measured throughout a reproductive season.

### 2.5 Analysis of plasma samples

We used a series of tests on the plasma separated from blood samples to assess individual immune function and stress physiology. For innate immune measures, we conducted an *Escherichia coli* bacteria‐killing assay to determine levels of resistance to infection. We conducted the assay according to a previously established protocol (French & Neuman‐Lee, 2012). We added plasma samples (6 μL) to sterile CO_2_‐Independent Media (Gibco© 16 μL) with l‐glutamine, mixed, and added to an *Escherichia coli* suspension (1 × 10^5^ CFU in 10× PBS; 5 μL) into each well of a 96‐well plate. We incubated the plate for 30 min at 37°C and added 125 μL of sterile tryptic soy broth to each well. We included both negative and positive controls. We used a microplate reader (BioRad xMark; Hercules, CA, USA) to read a baseline absorbance measurement for the plate at 300 nm. We incubated the plate at 37°C for 12 h and then read the absorbance a second time to evaluate bacterial growth. We calculated raw values by subtracting the baseline measurements from the measurements after 12 h of incubation. We then calculated final bacterial killing values as the percentage of the positive controls (where positives were considered 100% bacterial growth).

We measured plasma corticosterone levels via radioimmunoassays (RIAs) using a previously described and established protocol (Moore, 1986; Neuman‐Lee & French, 2014) and completed such assays in duplicate. Briefly, we added 30% ethyl acetate: isooctane mixture to plasma samples and snap‐froze them by immersion in dry ice and methanol. The supernatant was evaporated using nitrogen gas and resuspended in phosphate buffered saline. We calculated recoveries using a separate aliquot of these re‐suspended fractions which allowed for adjustment of the final sample concentration. Coefficients of variation for all assays were <20%.

Reactive oxygen metabolites in the plasma were measured to determine if there were elevated levels of oxidative stress. We followed the protocol included with the d‐ROMs test kit (Diacron, Grosseto, Italy). Briefly, we mixed the provided R_1_ and R_2_ reagents in a 1:100 dilution to create an acidic buffered solution with a chromogen. This resulting solution was kept in the dark until 5 μL of sample plasma was added into separate wells of a 96‐well microplate and 100 μL of the R_1_/R_2_ solution was added to each well. Nanopure water was used as sample blanks and the provided serum was used as a calibrator solution. We followed the “end‐point mode” from the manufacturer's protocol and measured absorbance at 505 nm after a 90 min incubation at 37°C. The resulting units are in mg H_2_O_2_/dL (1 CARR *U* = 0.08 mg H_2_O_2_/dL).

### 2.6 Female reproductive investment index

**FIGURE 1:**
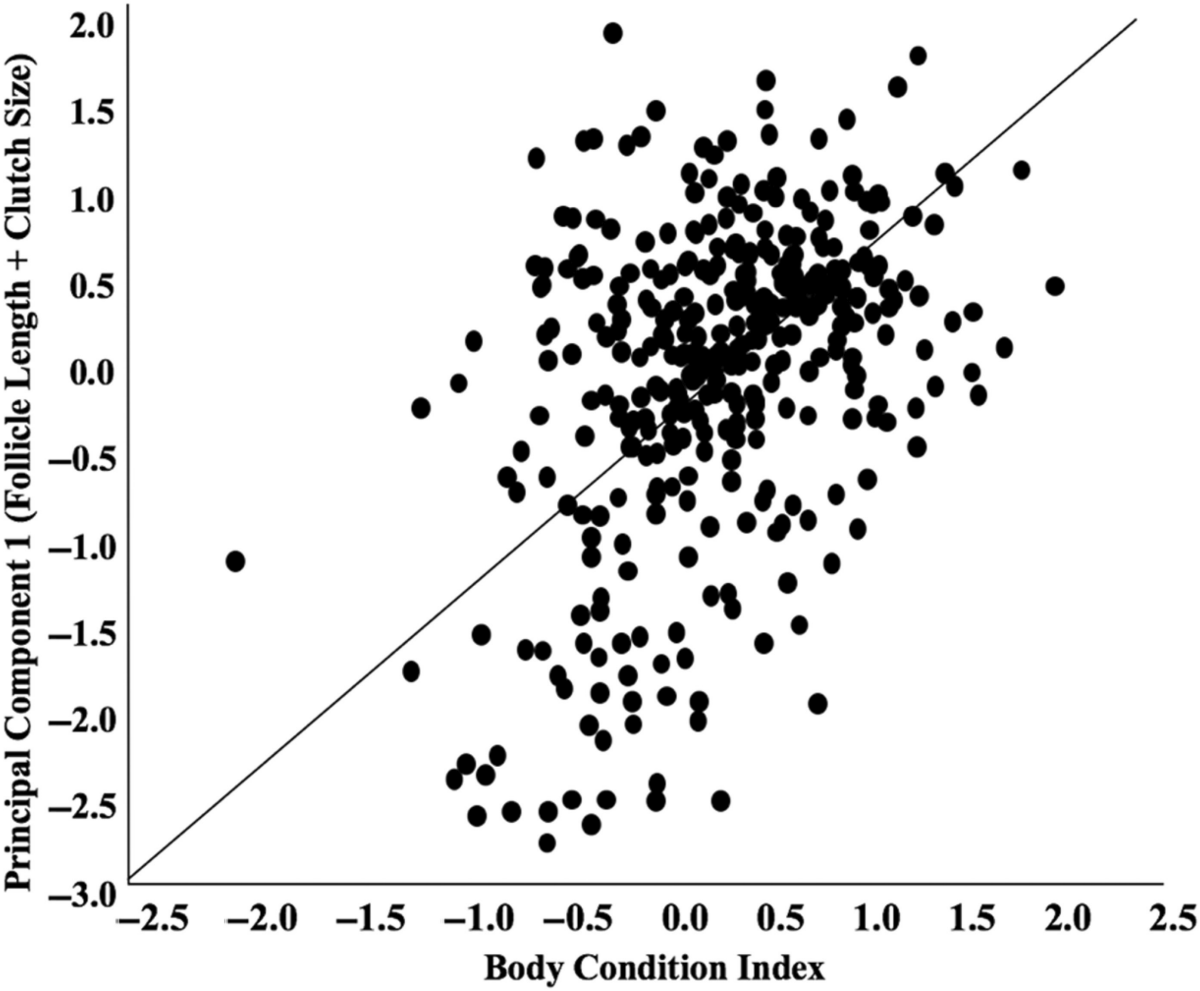
Regression of body condition index on principal component 1 from factor reduction of follicle length + clutch size. Residuals of this regression were used as the female reproductive investment index.

Body size and mass should correlate with reproductive potential in female lizards (Tinkle et al., 1970), so we considered variation in follicle size and clutch size relative to body condition to be a good indicator of differential investment in reproduction among females. We used principal component analysis to reduce factors, combining clutch size and follicle length, and a single principal component was extracted from this analysis (eigenvalue = 75.3%). To calculate the reproductive investment index for each female, we regressed this principal component on body condition and calculated the residuals, providing a reproductive index for each female (Figure 1). The female reproductive investment index was used as the dependent variable in our statistical models.

### 2.7 Male investment

Because body condition is a known factor in reproductive success in males in some lizard species (Hofmann & Henle, 2006; Salvador et al., 2008), we used body condition as the dependent variable in our models with a suite of environmental and physiological metrics as factors and covariates. We would anticipate context‐dependent differences in the relationships between body condition and the other blood metrics described above if males are adjusting investment in self versus investment in reproduction during their reproductive season.

### 2.8 Statistical analysis

All analyses were completed using SPSS (version 25.0; IBM, Inc.). Prior to analysis, dependent variables and covariates were checked for normal distribution. Corticosterone (CORT) and oxidative stress (d‐ROMs) were not normally distributed. Each of these variables was log‐transformed for the entire data set, after which they were normally distributed. Body condition index (BCI) and bacterial killing ability (BKA) were normally distributed.

For females, we used General Linear Mixed Models with reproductive investment index as the dependent variable, vitellogenic stage (stage: early, middle, late, gravid) as an ordered factor, precipitation (precip: low or high), and site type (site: rural or urban) as fixed factors, and body condition index (BCI), corticosterone (CORT), oxidative stress (d‐ROMs), and bacterial killing ability (BKA) as continuous covariates. Year and site were included as a random factor to statistically control for variation in sample sizes and unmeasured environmental variation among years. Lizard identity was included as a random effect to statistically control for incomplete replication of recaptured lizards among years. We checked for multicollinearity among covariates, and found no significant collinearity among the variables included in the models (BCI and CORT, *r* = .27; VIF = 1.96; BCI and d‐ROMs, *r* = .26, VIF = 1.90; CORT and d‐ROMs, *r* = .10, VIF = 1.1; BKA and CORT, *r* = .10, VIF = 1.96; BKA and BCI, *r* = .04, VIF = 1.04; BKA and d‐ROMs, *r* = .02, VIF = 1.02).

We used multi‐model inference with second‐order Akaike's Information Criterion (AIC_c_) for model selection, including additive models without interactions, models with our hypothesized 3‐way interactions, and an intercept‐only model. Body condition index and the random variables were included in all models. Overall, 42 models were assessed, from which we present the three most likely models and the global model (Table 2). The information criterion approach is appropriate for complex, observational, ecological studies by facilitating identification of the most likely, most informative model (Burnham & Anderson, 2002). AIC_c_ penalizes overparameterization, and models with the lowest AIC_c_ score are considered the best models. From the model with the lowest AIC_c_ value, each subsequent model can be evaluated by the change in AIC_c_ (∆AIC_c_) that accompanies the change in variables from the model with the lowest AIC_c_ score. This further permits the calculation of Akaike's weight (*w*_*i*_) for each model, which is the likelihood that the model is the best model. Even a set of bad models will still have a best model, so McFadden's pseudo‐*r* ^2^ was calculated for the models to evaluate the overall quality of the models. This can be indicated by the intercept‐only model having the lowest AIC_c_. An intercept‐only (null) model was also included in model selection. An AIC‐based approach was chosen over model averaging or evaluating the significance of every interaction because our goal was to attempt hypothesis testing instead of exploratory observation, while still identifying models simple enough to have some application for resource managers also using complicated datasets in variable systems.

**TABLE 2:** Model results from analysis of factors explaining reproductive investment in female *Uta stansburiana*.

Model	*K*	−2LL	AIC_c_	∆AIC_c_	*w*_*i*_
Precip*Site Type*d‐ROMs + Precip*Site Type*Stage + BCI	27	597.32	599.34	0	0.26
Precip*Site Type*Stage + BCI	26	598.58	600.60	1.26	0.14
Precip*Site Type*d‐ROMs + Precip*Site Type*Stage + BCI	27	598.64	600.66	1.32	0.11
Global Model (all 3‐way interactions + BCI)	109	652.93	654.96	55.61	0.000

*Note*: The explanatory variables contained within each model, the number of parameters (K), −2 Log Likelihood (−2LL), second‐order Akaike's information criterion values (AIC_c_), the difference between each model and the best model (∆AIC_c_), and the Akaike weight (*w*_*i*_), representing the likelihood of that model being the best model. The AIC_c_ values were generated from a general linear mixed model with female reproductive index as the dependent variable. The four best models and the global model are displayed.

For males, we used body condition index as the dependent variable. Other model parameters and analyses were as described above for females, but without body condition as a covariate (Table 2).

We calculated *r* ^2^ values to determine the strength of relationships between continuous variables and effect sizes (*η* ^2^) for comparisons of means among categorical variables.

## 3 RESULTS

The best model describing female reproductive investment included a three‐way interaction of precipitation*site type*d‐ROMs, a three‐way interaction of precipitation*site type*stage, and the individual variable of body condition index (Table 2). This model was marginally more likely to be the best model (*w*_*i*_ = 0.26) than other models and had a McFadden's pseudo‐*r* ^2^ of 0.19. Corticosterone and bacterial killing ability were not involved in any of the hypothesized interactions with site type or precipitation.

### 3.1 Females

**FIGURE 2:**
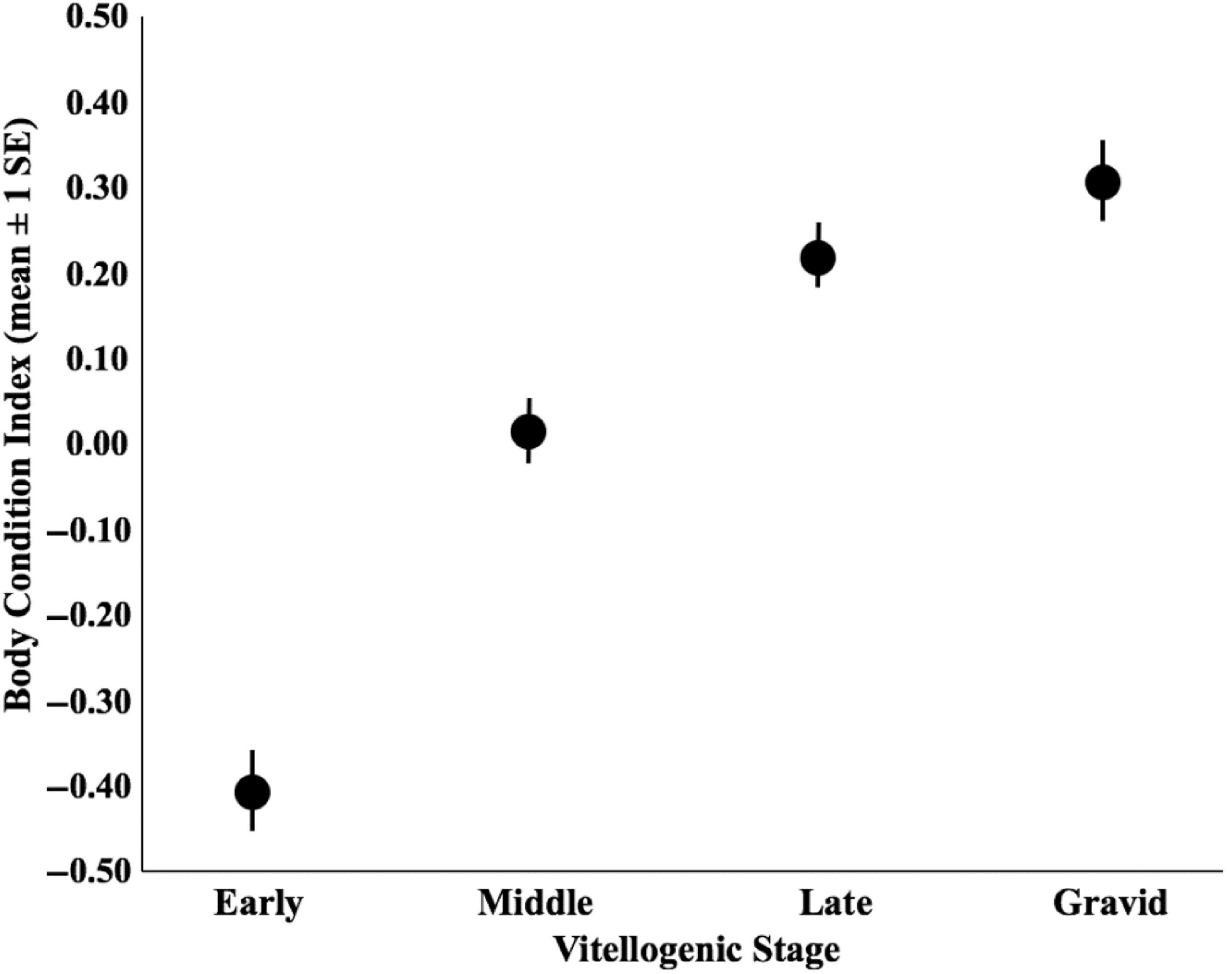
Differences in body condition among vitellogenic stages in female *Uta stansburiana*.

**FIGURE 3:**
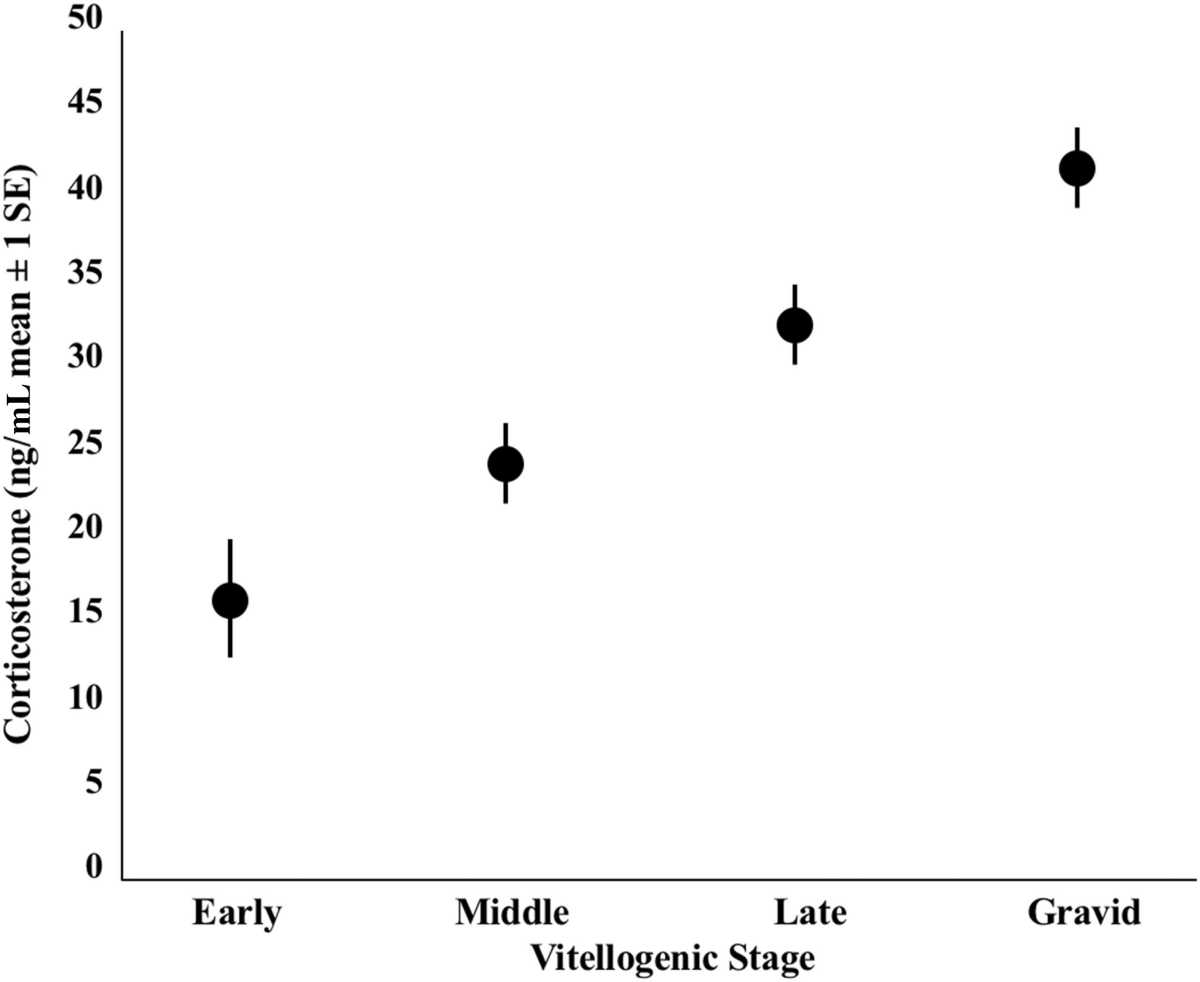
Differences in corticosterone among vitellogenic stages in female *Uta stansburiana*.

**FIGURE 4:**
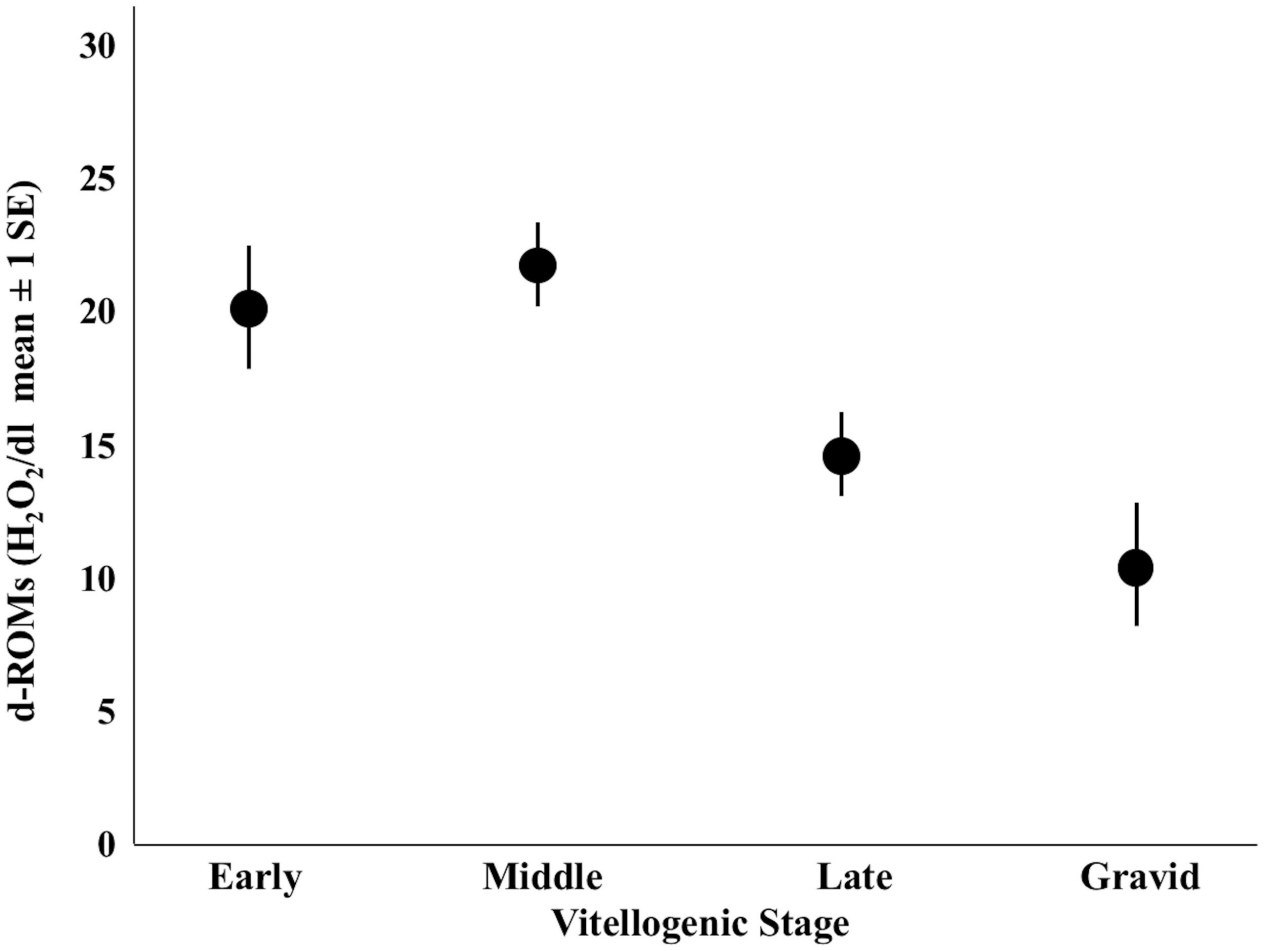
Differences in d‐ROMs among vitellogenic stages in female *Uta stansburiana*.

There were substantial differences among female vitellogenic stages with respect to body condition index (Figure 2; *η* ^2^ = 0.268). Tukey's post hoc test revealed that early stage lizards were in significantly lower body condition than all other stages (*p* < .001 in all cases), middle stage lizards were in significantly lower body condition than each late stage and gravid lizard (*p* < .001 in both cases), and there was no significant difference in body condition between late stage and gravid females (*p* = .667). There were also significant differences in corticosterone among stages (Figure 3; *η* ^2^ = .164). Tukey's post hoc test revealed that early stage lizards had significantly lower corticosterone than each of the other stages (*p* < .001 in all cases), that middle stage lizards had significantly lower corticosterone than gravid lizards (*p* = .022), and that there were no other significant pairwise differences (*p* < .234 in all cases). There were also significant differences in d‐ROMs among females in different stages (Figure 4; *η* ^2^ = 0.115). Tukey's post‐hoc test revealed that early stage lizards had significantly greater d‐ROMs than gravid lizards (*p* = .003), middle stage lizards had significantly greater d‐ROMs than late stage lizards (*p* = .011) and gravid lizards (*p* < .001). There were no other significant pairwise differences (*p* > .061 in all cases).

The relationship between d‐ROMs and reproductive index differed among different site types, and revealed context‐dependent relationships with precipitation. This is indicated by the presence of the three‐way interaction of precipitation*site type*d‐ROMs in the best model (figure not shown). Specifically, there was a weak positive, linear relationship (*r* ^2^ = .07) in lizards at rural sites in low precipitation years, and a very weak negative linear relationship (*r* ^2^ = .002) in lizards at urban sites in low precipitation years. The importance of the interaction term is that in high precipitation years, there is a much stronger positive linear relationship in urban lizards (*r* ^2^ = .116), which is the opposite of the low precipitation years; whereas, the positive linear relationship between d‐ROMs and reproductive index in rural lizards is not substantially different (*r* ^2^ = .013).

**FIGURE 5:**
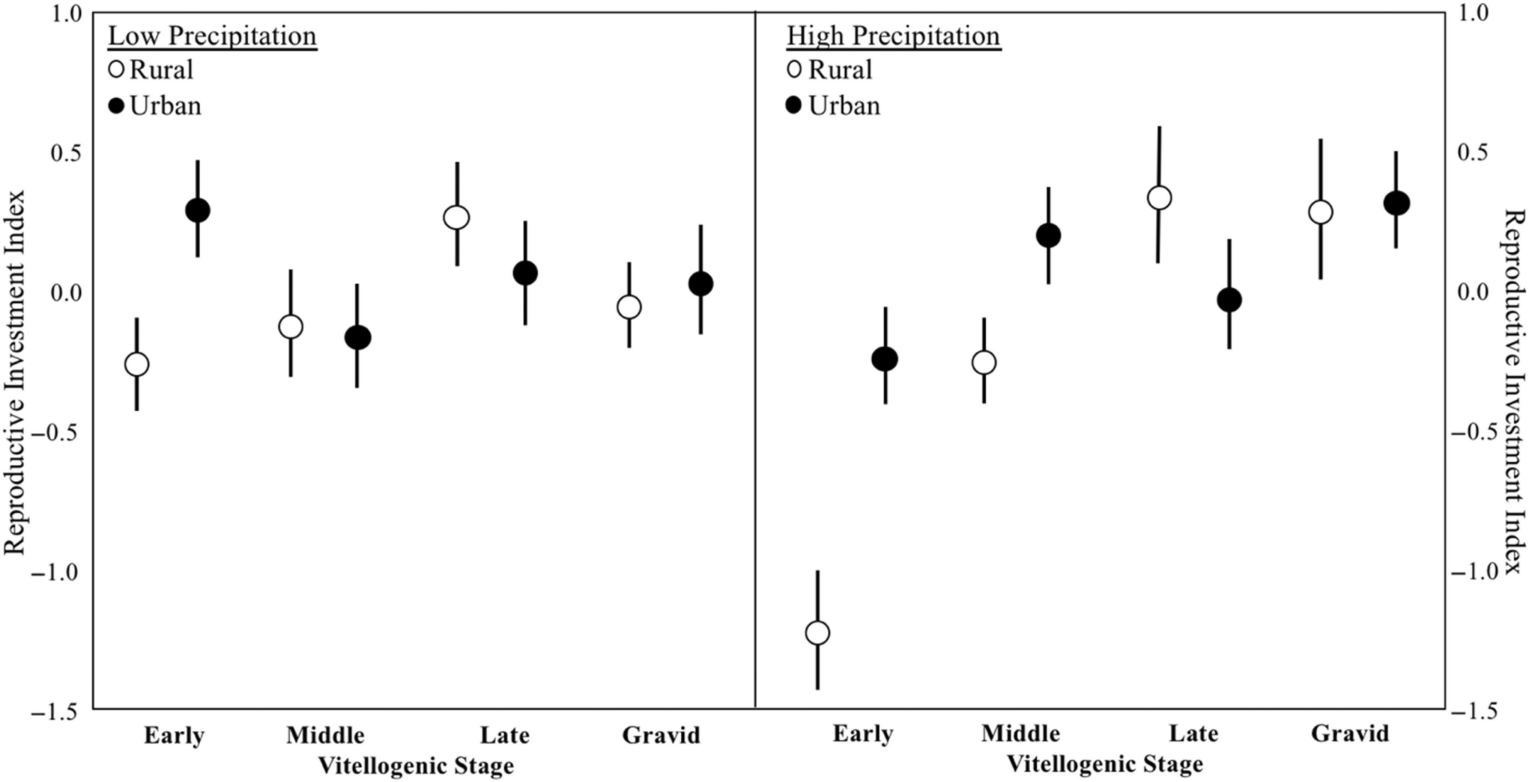
Differences in reproductive investment among different vitellogenic stages in rural and urban sites in years with low precipitation and high precipitation. The circles and bars represent the means plus or minus 1 standard error.

Differences in reproductive investment associated with precipitation levels were influenced by the degree of anthropogenic development of the habitat, but these differences varied by vitellogenic stage (precipitation*site type*stage; Figure 5). In low precipitation and in high precipitation years, urban lizards invested more in reproduction during the early vitellogenic stages compared to the rural lizards, but in low precipitation years, that investment dropped in urban lizards while it increased in rural lizards (Figure 5). Further, in both low precipitation and high precipitation conditions, rural lizards invested more into reproduction in the late vitellogenic stage.

**FIGURE 6:**
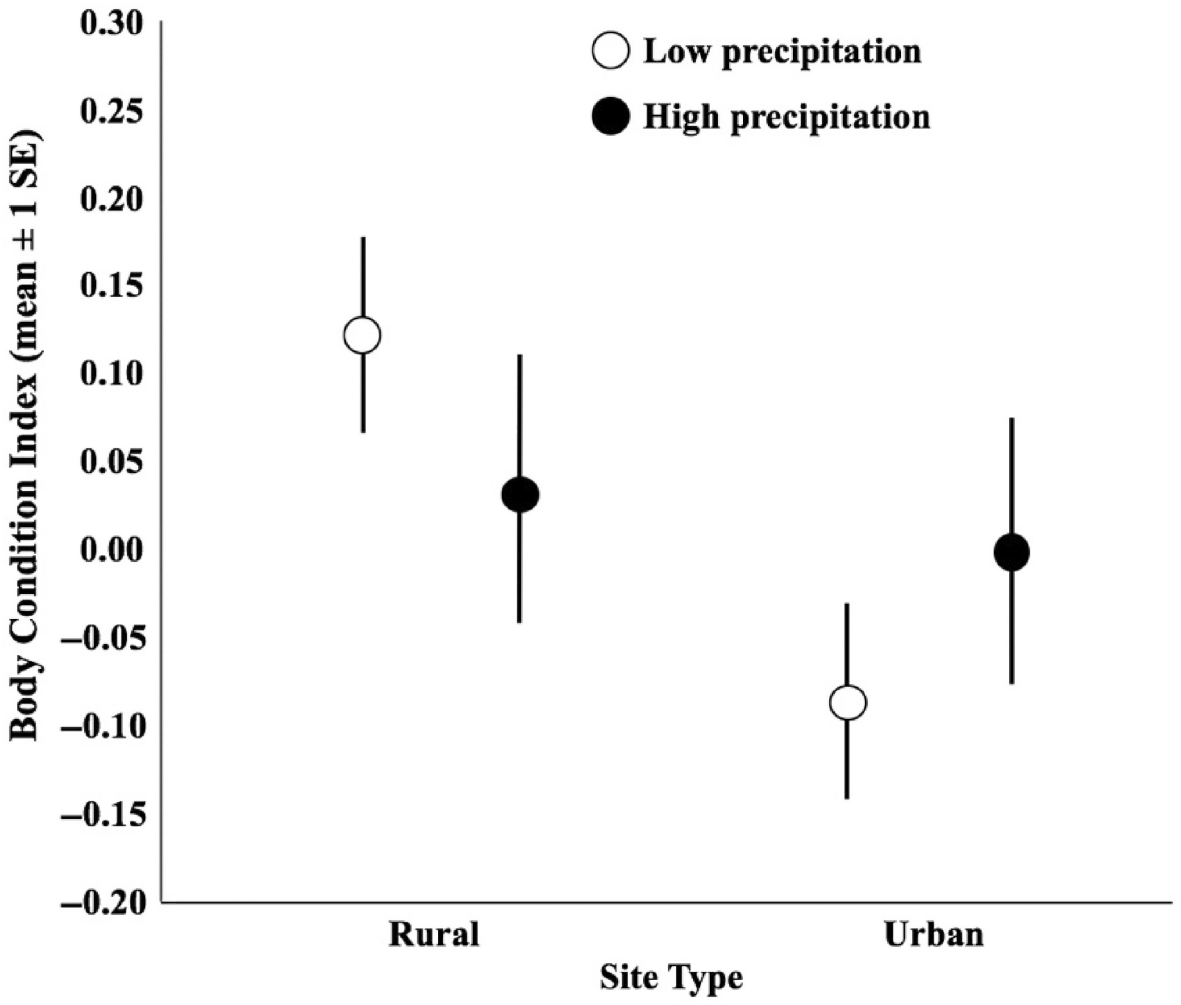
Differences in body condition of female *Uta stansburiana* in years with high and low precipitation at urban and rural sites.

Differences in body condition associated with precipitation levels were also influenced by the degree of anthropogenic development (high precipitation *η* ^2^ = 0.05, low precipitation *η* ^2^ = 0.15; Figure 6).

### 3.2 Males

**FIGURE 7:**
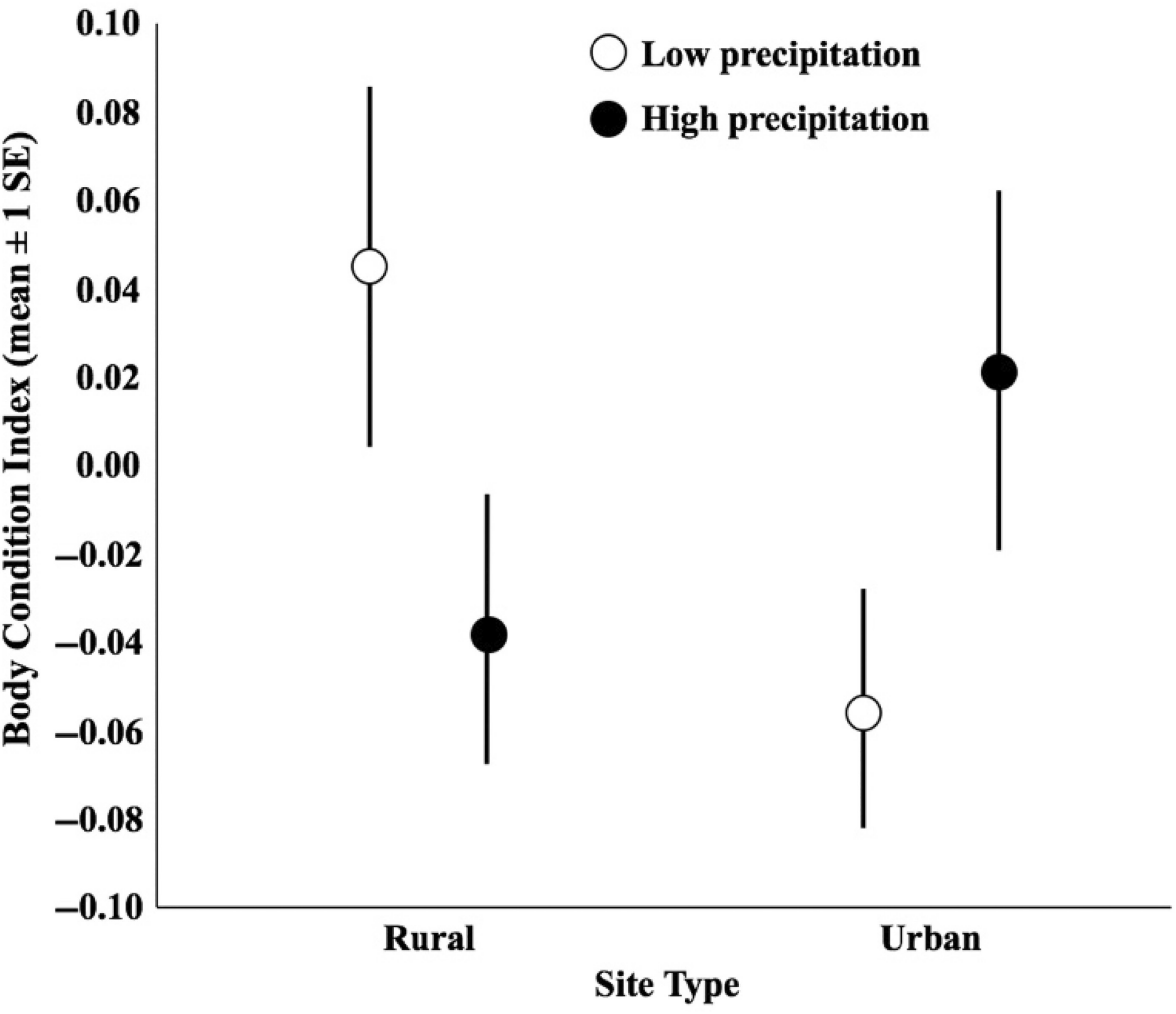
Differences in body condition of male *Uta stansburiana* in years with high and low precipitation at urban and rural sites.

For the analysis of male investment patterns, the top five models each explained very little variance in male body condition, as the best model had a McFadden's pseudo‐*r* ^2^ of only .034. The best model describing male body condition included a precipitation by site interaction and the main effects of precipitation and site (*w*_*i*_ = 0.256, Table 3). In high precipitation years, there was no difference in body condition between males at urban and rural sites (*η* ^2^ = 0.09), but in low precipitation years, males at rural sites were in better condition than those at urban sites (*η* ^2^ = 0.22; Figure 7). This model was not substantially better than the second model, with the aforementioned variables and corticosterone levels (*w*_*i*_ = 0.228, Table 3); however, regression of body condition index and corticosterone revealed a weak inverse relationship with an *r* ^2^ of only .019, further confirming that variation in male body condition is not strongly related to the suite of variables explored here.

**TABLE 3:** Model results from analysis of factors explaining body condition in male *Uta stansburiana*.

Model	*K*	‐2LL	AIC_c_	∆AIC_c_	*w*_*i*_
Precip*Site + Precip + Site	5	311.56	313.57	0	0.256
Precip*Site + Precip + Site + CORT	6	312.46	314.47	0.90	0.228
Precip + Site	4	314.71	316.73	3.15	0.140
Precip*Site + Site*CORT + Precip + Site + CORT	7	315.09	317.11	3.53	0.125
Precip*Site + Precip + Site + CORT + d‐ROMs	8	315.56	317.58	3.55	0.124
Global Model (all variables and interactions)	33	442.17	444.19	130.62	0.000

*Note*: The model variables, the number of parameters (*K*), −2 Log Likelihood (−2LL), second‐order Akaike's information criterion values (AIC_c_), the difference between each model and the best model (∆AIC_c_), and the Akaike's weight (*w*_*i*_), representing the likelihood of that model being the best model. The AIC_c_ values were generated from a general linear mixed model with female reproductive index as the dependent variable. The five best models and the global model are displayed.

## 4 DISCUSSION

### 4.1 Overview of findings

After 6 years of evaluating side‐blotched lizards in urban and rural populations, we found evidence that both individual and environmental factors contribute to successful reproductive investment. Moreover, there were complex interactions among individual, environmental, and temporal characteristics leading to variable reproductive investment. Specifically, we found that body condition and corticosterone increased throughout vitellogenesis, and oxidative damage markers decreased. However, these patterns shifted significantly across both space (urbanization) and time (annual precipitation), with rural animals increasing reproductive output during rainier years compared to the more environmentally buffered urban populations, and individual characteristics being more important for females than males. We also found that the energetic allocations allowing for greater reproductive effort in rural lizards occurred early in the vitellogenic process.

### 4.2 Individual characteristics and interactions

Our study demonstrates that physiological indices can vary within a reproductive event (i.e., across vitellogenesis). Body condition indices (BCI) increased across vitellogenesis and were highest at gravidity. Body mass tends to increase across vitellogenesis due to the increase in follicle size and mass during the reproductive cycle, so this finding was expected. Because body mass is integral to calculating BCI, it is likely that developing follicles and shelled eggs at least partly contributed to this finding. However, body condition prior to reproduction can predict reproductive success (Naulleau & Bonnet, 1996), parturition date (Bêty et al., 2003) and litter or clutch size (Bêty et al., 2003; Lourdais et al., 2003; Risch et al., 2007). Hence, it is possible that the increase in BCI throughout vitellogenesis was a result of robust females beginning their reproductive efforts in a condition better suited to yolking more or larger follicles that, in turn, contributed to increased mass and BCI. Essential nutrients like proteins, lipids, and carbohydrates must be mobilized for a successful clutch, and how and when these nutrients are allocated also depends on internal constraints such as existing resource stores in the body. For instance, Durso et al. (2020) found that female side‐blotched lizards with greater toe carbon:nitrogen ratios were able to mount larger corticosterone responses in response to experimental restraint stress. Considering higher carbon:nitrogen ratios were also linked with greater body condition, this suggests that animals with higher BCIs simply had more available energy and resources to respond. This sort of resource allocation also depends on reproductive stage. Under controlled laboratory conditions using ^15^N‐leucine, female side‐blotched lizards invested more protein into self‐maintenance (in the form of wound healing) early in reproduction, but animals in later reproductive stages invested more into follicles (Durso & French, 2018). In a related study using ^15^N‐leucine and ^13^C‐1‐palmitic acid (to determine lipid mobilization), Pettit et al. (2019) found similar allocations of protein and fats into follicles at later reproductive stages, with self‐maintenance allowances occurring earlier. Altogether, these results support our observation that body condition and reproductive stage are critical to consider when assessing energy and resource mobilization in response to external stressors.

Similar to BCI, circulating corticosterone was highest at gravidity and lowest in early vitellogenesis. Corticosterone, which aids in the allocation of energetic resources towards physiological functions, is typically elevated during periods of high energetic investment, such as reproduction (Brusch IV et al., 2020; Moore & Jessop, 2003; Woodley & Moore, 2002). Comparisons of corticosterone levels between vitellogenic and gravid lizards have shown that gravid females have higher baseline corticosterone compared to vitellogenic lizards (Woodley & Moore, 2002), and that corticosterone may aid or be involved in nesting behaviors and oviposition in reptiles (Anderson et al., 2014; Rubenstein & Wikelski, 2005; Tyrrell & Cree, 1998). Indeed, baseline corticosterone increased across the breeding season and peaked during nesting season in female Galapagos marine iguanas (*Amblyrhynchus cristatus*) (Rubenstein & Wikelski, 2005). Therefore, the increase in corticosterone observed in gravid side‐blotched lizards could be explained by their preparation to oviposit, following previously observed trends. Despite increases in corticosterone across vitellogenesis and into gravidity, gravid females may exhibit a dampened response to external stressors. For example, gravid tree lizards (*Urosaurus ornatus*) did not mount an elevated corticosterone response to handling restraint, unlike vitellogenic conspecifics (Woodley & Moore, 2002) and gravid tuatara (*Sphenodon punctatus*) also exhibited dampened responses to a stress challenge (Anderson et al., 2014). Although empirical data are lacking, this could be due to reduced energy available due to investment in reproduction, or to suppression of a stress response in order to maintain current reproductive activities (Jessop et al., 2000), an idea that should be investigated in future studies.

Oxidative stress was highest during early vitellogenesis and lowest in gravid individuals, which suggests elevation of metabolism and yolk precursors in early‐vitellogenic females could be the cause of higher oxidative stress. During vitellogenesis, egg yolk precursors derived in the liver begin circulating in the blood and are deposited into oocytes (Price, 2017), mirroring high levels of energy metabolites (Webb et al., 2019) and vitellogenin (Lindsay et al., 2020) early in reproduction. Considering that reproduction is physiologically stressful, free‐living lizards are likely able to handle vitellogenic investment differently based on their condition during the breeding season. Reproduction is known to upregulate metabolism in reptiles (DeMarco, 1993; Dupoué & Lourdais, 2014; Foucart et al., 2014; Van Dyke & Beaupre, 2011), which can generate disproportionate amounts of reactive oxygen metabolites, but see Costantini et al. (2014).

If oxidative stress is directly related to energetic investment into reproduction, then lower levels of oxidative stress in late‐vitellogenic and gravid females in our study may be due to reduced circulating yolk proteins and lipids because yolk investment is minimal after ovulation. Alternatively, lipids and reactive oxygen species me be deposited in the yolking follicles, thereby reducing circulating levels. For example, vitellogenin and oxidative stress levels were lower in post‐ovulatory females than vitellogenic female painted dragon lizards due to deposition in the follicles (*Ctenophorus pictus*) (Lindsay et al., 2020). Alternatively, but not exclusively, lower levels of oxidative stress may be due to “oxidative shielding,” whereby oxidative damage is reduced in gravid females to protect gametes or developing offspring (Blount et al., 2016; Webb et al., 2019). Emerging evidence suggests that vitellogenin may have antioxidant effects that counteract the oxidative costs of reproduction (Lindsay et al., 2020) as indicated by sustained levels of vitellogenin in post‐ovulatory female painted dragon lizards, when yolking of follicles has ceased.

Although we observed stage‐dependent reproductive patterns across individuals, there was a high degree of variability among individuals that was related to differences in reproductive investment.

### 4.3 Environmental factors and interactions

We found that multiple extrinsic factors interacted to impact reproduction. Rainier years had a larger effect on the reproductive investment of rural populations than their urban counterparts, which appeared to be more buffered against environmental constraints. This could be because the urban populations are situated near residential areas and golf courses that received regular irrigation to care for landscaping, blunting the effect of low precipitation. Anthropogenic watering has been shown to provide sanctuary to some species during otherwise dangerous droughts (Roe et al., 2011; Waite et al., 2007). Many species, termed urban exploiters, do well in anthropogenically altered landscapes for multiple reasons, as shown by Ackley et al. (2015). Some unique urban factors like artificial lighting can be beneficial for the wildlife that can use it to their advantage, as seen in bats (Schoeman, 2016) and *Anolis* lizards (Thawley & Kolbe, 2020). Many of the species that can best take advantage of these disturbed situations are invasive (Colléony & Shwartz, 2020; Hall & Warner, 2017) or highly generalist, and thus resilient to dynamic landscapes (Ducatez et al., 2018). Side‐blotched lizards are a generalist species (Hibbitts et al., 2013), and are good candidates to thrive under these conditions.

In wetter years, rural populations not only invested more into reproduction than in drier years, but also had greater reproductive investment than urban populations experiencing the same above‐average precipitation. There are multiple possible explanations for this observation. The terminal investment hypothesis (Williams, 1966) suggests that the internal struggle between immediate and lifetime fitness is reduced when death is imminent, and it has been supported by multiple studies (Creighton et al., 2009; Descamps et al., 2007; Velando et al., 2006). However, if rural animals lack the anthropogenic buffering of their urban counterparts and suffer more during dry years, one would predict higher reproductive investment in drought years in rural populations to maximize lifetime fitness, the opposite of what was observed. Additionally, an important intrinsic cue for terminal investment is the age of the animal, and critical extrinsic cues are parasite and pathogen infection (Duffield et al., 2017). Based on population models, the rural populations have greater average survival (Lucas & French, 2012), further indicating that the terminal investment hypothesis cannot explain our findings. It is also possible that even during the drier years of this study, the drought was not severe enough to elicit terminal investment in any of the populations observed. Indeed, when severe, prolonged droughts are sufficient to halt the breeding season in other populations of this species, there is a subsequent decrease in body condition in females (but not males) that could indicate an attempt at terminal investment (Zani & Stein, 2018), which we did not observe. Instead, in drier years, rural animals maintained better body condition than urban ones, which may be due to lower relative investment into reproduction during dry years, which might allow rural animals to save resources to invest into reproduction during wet years.

There could also be behavioral differences in the populations in terms of resource use. Smith et al. (2019) found that side‐blotched lizards from northern populations, where they face harsher winters and greater abiotic stress, consumed more food during a common garden study than southern lizards, perhaps because they are better suited to take advantage of fleeting resources. If the rural populations in this study faced greater resource fluctuation than the urban populations, they could have been compensating in a similar way. This would explain the better body condition in rural animals, even in drier years, and their capacity to take advantage of increased resources during wet years. Zani (2008) observed that larger females had earlier first clutches than smaller females in the northern part of their range, indicating that large size and high body condition might be favored in harsher or more variable environments. Based on previous work showing greater survival at rural sites (Lucas & French, 2012), it is likely that the older age structure and greater average body condition in the rural populations allowed them to draw more on stored resources for reproduction in drier years.

Vitellogenic stage played different roles in urban and rural populations in relation to precipitation and body condition, with urban lizards seemingly better buffered from annual precipitation effects than rural ones. In drier years, rural animals at earlier vitellogenic stages invested more into reproduction, but only if they had high body condition, whereas urban animals invested more consistently across years. This could be explained by more stable hydric conditions at the urban sites, or by rural animals being better able to postpone immediate reproduction to optimize lifetime fitness. Altogether, these results suggest shifts in life‐history strategy across a rapidly urbanizing landscape, with annual or seasonal adjustments being made in response to varying precipitation and the food resources that fluctuate with it.

## 5 CONCLUSIONS

The complex drivers and limitations of reproduction in free‐living animals are not yet clear, although the importance of understanding them for conservation and basic science could not be clearer. We integrate data from several physiological systems that are particularly important for animals making reproductive decisions and completing the reproductive process. These same systems also appear important in dictating annual differences in reproduction that arise through environmental and climate interactions, but there are certainly additional factors to consider. In particular, the interaction of extrinsic and intrinsic factors can lead to disparate reproductive strategies in even closely spaced populations. We recommend that future studies investigate other physiological interactions with respect to unmeasured internal (stored energy) and external (parasitism, competition) stressors, while still focusing on reproductive/recruitment outcomes as critical fitness measures. More broadly, we suggest that researchers acknowledge the complexity of their systems when asking complicated questions, and understand that a single field season of sampling, while intrinsically valuable, has limited power to address larger questions related to individual responses to changing environments. More funding resources and attention are required to fully elucidate the nuances occurring now, as the planet is changing at an increasing pace.

## AUTHOR CONTRIBUTIONS

**Geoffrey D. Smith:** Conceptualization (equal); data curation (equal); formal analysis (equal); funding acquisition (supporting); investigation (lead); methodology (equal); project administration (supporting); resources (supporting); supervision (supporting); visualization (supporting); writing – original draft (lead); writing – review and editing (lead). **Travis E. Wilcoxen:** Data curation (equal); formal analysis (lead); writing – original draft (supporting); writing – review and editing (equal). **Spencer B. Hudson:** Investigation (equal); writing – original draft (supporting); writing – review and editing (supporting). **Emily E. Virgin:** Investigation (supporting); writing – original draft (supporting); writing – review and editing (supporting). **Andrew M. Durso:** Data curation (equal); investigation (lead); methodology (equal); project administration (equal); supervision (equal); writing – original draft (supporting); writing – review and editing (supporting). **Marilize Van der Walt:** Investigation (supporting); writing – original draft (supporting); writing – review and editing (supporting). **Austin R. Spence:** Investigation (supporting); writing – original draft (supporting); writing – review and editing (supporting). **Lorin A. Neuman‐Lee:** Investigation (supporting); writing – original draft (supporting); writing – review and editing (supporting). **Alison C. Webb:** Data curation (supporting); investigation (supporting); writing – original draft (supporting); writing – review and editing (supporting). **Patricia A. Terletzky:** Data curation (equal); formal analysis (equal); writing – original draft (supporting); writing – review and editing (supporting). **Susannah S. French:** Conceptualization (lead); data curation (equal); formal analysis (equal); funding acquisition (lead); investigation (supporting); methodology (equal); project administration (lead); resources (lead); supervision (lead); visualization (lead); writing – original draft (supporting); writing – review and editing (supporting).

## CONFLICT OF INTEREST STATEMENT

There are no conflicts of interests, financial or otherwise, for any of the authors.

## Data Availability

Data available from the Utah State University Digital Commons: https://digitalcommons.usu.edu/; https://doi.org/10.26078/1k4b‐ar74.
